# Antimicrobial and Antioxidant Activities of *N*-2-Hydroxypropyltrimethyl Ammonium Chitosan Derivatives Bearing Amino Acid Schiff Bases

**DOI:** 10.3390/md20020086

**Published:** 2022-01-19

**Authors:** Jingmin Cui, Xia Ji, Yingqi Mi, Qin Miao, Fang Dong, Wenqiang Tan, Zhanyong Guo

**Affiliations:** 1 Key Laboratory of Coastal Biology and Bioresource Utilization, Yantai Institute of Coastal Zone Research, Chinese Academy of Sciences, Yantai 264003, China; jmcui@yic.ac.cn (J.C.); yqmi@yic.ac.cn (Y.M.); qmiao@yic.ac.cn (Q.M.); fdong@yic.ac.cn (F.D.); wqtan@yic.ac.cn (W.T.); 2 University of Chinese Academy of Sciences, Beijing 100049, China; 3 Center for Ocean Mega-Science, Chinese Academy of Sciences, Qingdao 266071, China; 4 School of Pharmacy, Qilu Medical University, Zibo 255300, China

**Keywords:** *N*-2-hydroxypropyltrimethyl ammonium chloride chitosan, amino acid Schiff bases, antimicrobial activity, antioxidant activity

## Abstract

*N*-2-hydroxypropyltrimethyl ammonium chloride chitosan (HACC), a cationic quaternary ammonium salt polymer exhibiting good solubility in water, is widely used because of its low toxicity and good biocompatibility. Herein, through ion exchange reaction, we prepared *N*-2-hydroxypropyltrimethyl ammonium chitosan derivatives bearing amino acid Schiff bases with good biological activities. The accuracy of the structures was verified by FT-IR and ^1^H NMR. The antibacterial activity, antifungal activity, and scavenging ability of DPPH radical and superoxide radical of HACC derivatives were significantly improved compared with that of HACC. In particular, HACGM (HACC-potassium 2-((2-hydroxy-3-methoxybenzylidene)amino)acetate) and HACGB (HACC-potassium 2-((5-bromo-2-hydroxybenzylidene)amino)acetate) showed good inhibitory effect on bacteria and fungi, including *Staphylococcus aureus*, *Escherichia coli*, *Botrytis cinerea,* and *Fusarium oxysporum* f. sp. *cubense*. The inhibition rate of HACGB on *Staphylococcus aureus* and *Escherichia coli* could reach 100% at the concentration of 0.1 mg/mL, and the inhibition rate of HACGM and HACGB on *Botrytis cinerea* and *Fusarium oxysporum* f. sp. *cubense* could also reach 100% at the concentration of 0.5 mg/mL. Improving antimicrobial and antioxidant activities of HACC could provide ideas and experiences for the development and utilization of chitosan derivatives.

## 1. Introduction

In the field of agriculture, the presence of bacteria and fungi in nature has a significant impact on the growth and reproduction of crops and livestock [1,2]. In order to avoid the damage and loss caused by plant pathogenic fungi, the synthesis and utilization of new fungicides are critical as drug resistance increases [3,4]. Moreover, bacterial infection in agricultural production process should not be underestimated, if in bacteria-infected animals and -polluted irrigation water, it will spread through the circulation and affect food security [1,5]. Chitin is the second largest polysaccharide after cellulose on Earth, widely found in arthropods (shrimps and crabs), mollusks (squids and snails), annelids (earthworms and hornworms), and mushrooms [6,7]. Chitosan, obtained by hydrolyzing the *N*-alkyl ethanamide from the chitin, is the only natural basic polysaccharide [8] and it has certain antibacterial and antifungal activities decided by its molecular weight, degree of deacetylation, temperature, pH of solution, species of tested fungi and bacteria, etc. [9,10]. Studies showed that chitosan could make the outer membrane of bacteria zigzagged, so that the permeability of the inner membrane increased, leading to the destruction of the integrity of the membrane eventually [11]. It was widely accepted that the permeability change of the cell membrane was caused by the combination of the positive amino ions in the chitosan solution and the negatively charged lipopolysaccharide on the surface of the cell membrane [12].

In recent years there have been significant advances in chitosan derivatives [13,14,15]. The synthesis and application of *N*-2-Hydroxypropyltrimethyl ammonium chloride chitosan (HACC) is a good example. HACC is synthesized by a reaction of chitosan and 3-chloro-2-hydroxypropyl trimethyl ammonium chloride (CHPTMAC) or glycidyl trimethylammonium chloride (GTMAC) [16,17]. Because of its good water solubility, safety, and non-toxicity, various applications of HACC and its derivatives have been studied such as delivery vehicles for live, attenuated vaccine, DNA vaccine, and inactivated vaccine [18,19,20]; adsorbents used to remove reactive dye or impurity ions from aqueous solutions [21,22]; inhibitor on mild steel [23]; and straw paper for antibacterial food-packaging [24]. However, most of the experimental data showed that the antimicrobial and antioxidant activities of HACC needed to be improved [25,26].

Schiff base is a compound containing imine or azomethine groups, which is synthesized by primary amines reacting with aldehydes or ketones [27]. Since the C=N double bond of a Schiff base contains lone pair electrons and has strong coordination properties, there are many studies on its metal complexes [28,29,30]. After the chitosan-Schiff base ligands and its metal complexes were synthesized, the antimicrobial activities of *Bacillus subtilis*, *Staphylococcus aureus*, *Escherichia coli*, *Klebsiella pneumonia,* and *Pseudomonas aeruginosa* were also tested [31]. An amino acid Schiff base ligand and its related copper (II) showed inhabitation towards the growth of *Escherichia coli*, *Bacillus subtilis*, and *Candida albicans* [32]. A cellulose-based Schiff base-Cu (II) complex with strong antibacterial activity against *Escherichia coli* and *Staphylococcus aureus* was synthesized [33]. Furthermore, many articles documented the synthesis of amino acid Schiff bases, which had the ability to inhibit the growth of bacteria and fungi [34]. In the synthesis of Schiff bases, the addition of potassium hydroxide or sodium hydroxide could promote the reaction in anhydrous ethanol or methanol solution; it also helped the dissolution of amino acids. The Schiff bases produced by the reaction were in the form of carboxylic acid salt; therefore, in this work, cationic chitosan quaternary ammonium salt was selected to combine with glycine Schiff bases through electrostatic action in an attempt to increase the application range of chitosan quaternary ammonium salt [35,36].

In this paper, a series of *N*-2-hydroxypropyltrimethyl ammonium chitosan derivatives bearing amino acid Schiff bases (HACGM, HACGB, HACGD, HACGS) were synthesized by the ion exchange reaction between HACC and potassium 2-((2-hydroxy-3-methoxybenzylidene)amino)acetate, potassium 2-((5-bromo-2-hydroxybenzylidene)amino)acetate, potassium 2-((3,5-dichloro-2-hydroxybenzylidene)amino)acetate, and potassium 2-((2-hydroxybenzylidene)amino)acetate, respectively, and their structures were characterized by Fourier transform infrared (FT-IR) spectroscopy and ^1^H Nuclear magnetic resonance (^1^H NMR) spectroscopy. Meanwhile, their antibacterial activity against *Staphylococcus aureus* (*S. aurous*) and *Escherichia coli* (*E. coli*) and antifungal activity against *Botrytis cinerea* (*B. cinerea*) and *Fusarium oxysporum* f. sp. *cubense* (*Foc*) and the scavenging ability of superoxide radical and DPPH-radical were also measured in this work.

## 2. Results and Discussion

### 2.1. Chemical Synthesis and Characterization

**Scheme 1:**
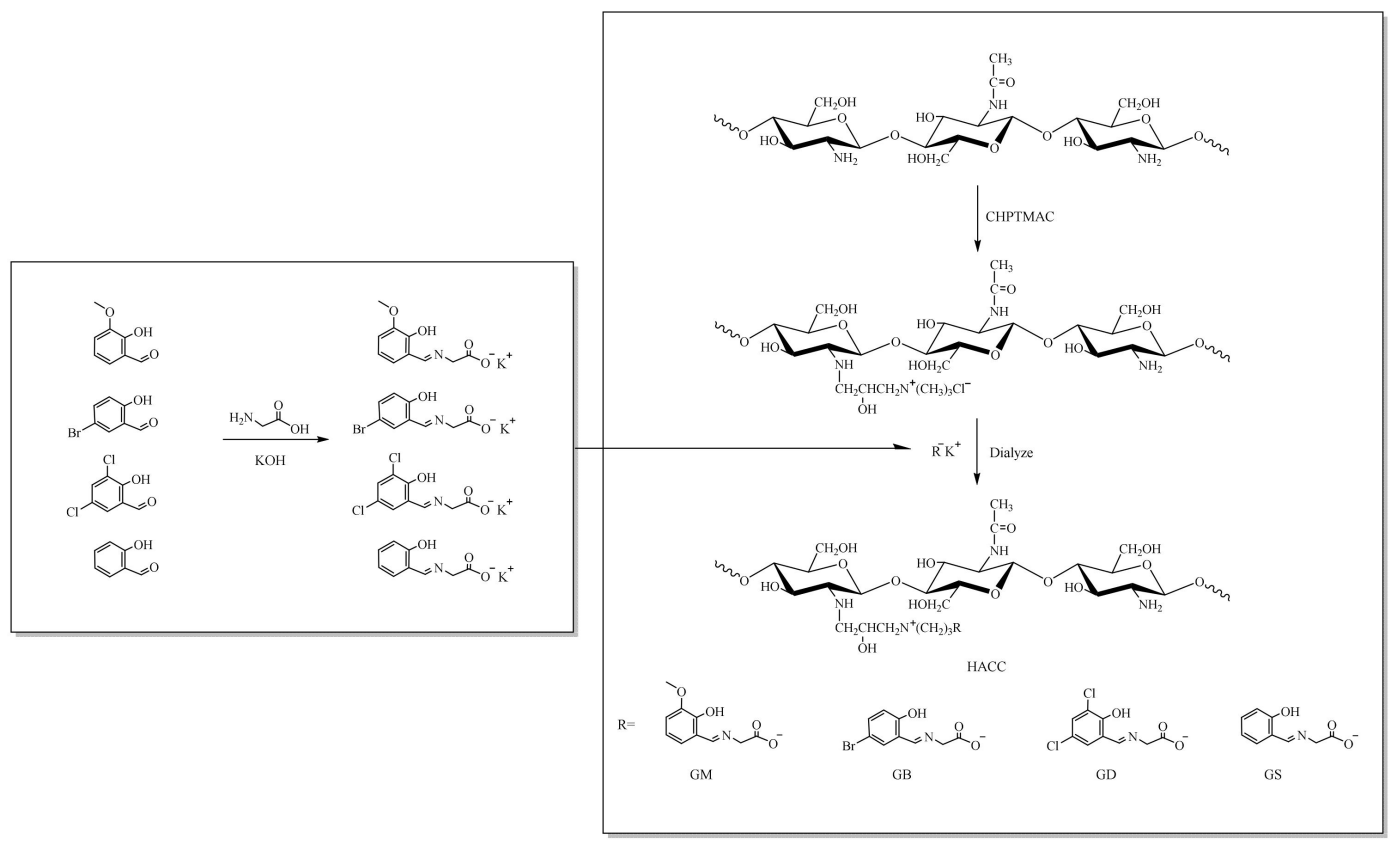
Synthesis pathway of chitosan derivatives of HACC and HACC derivatives.

**Figure 1:**
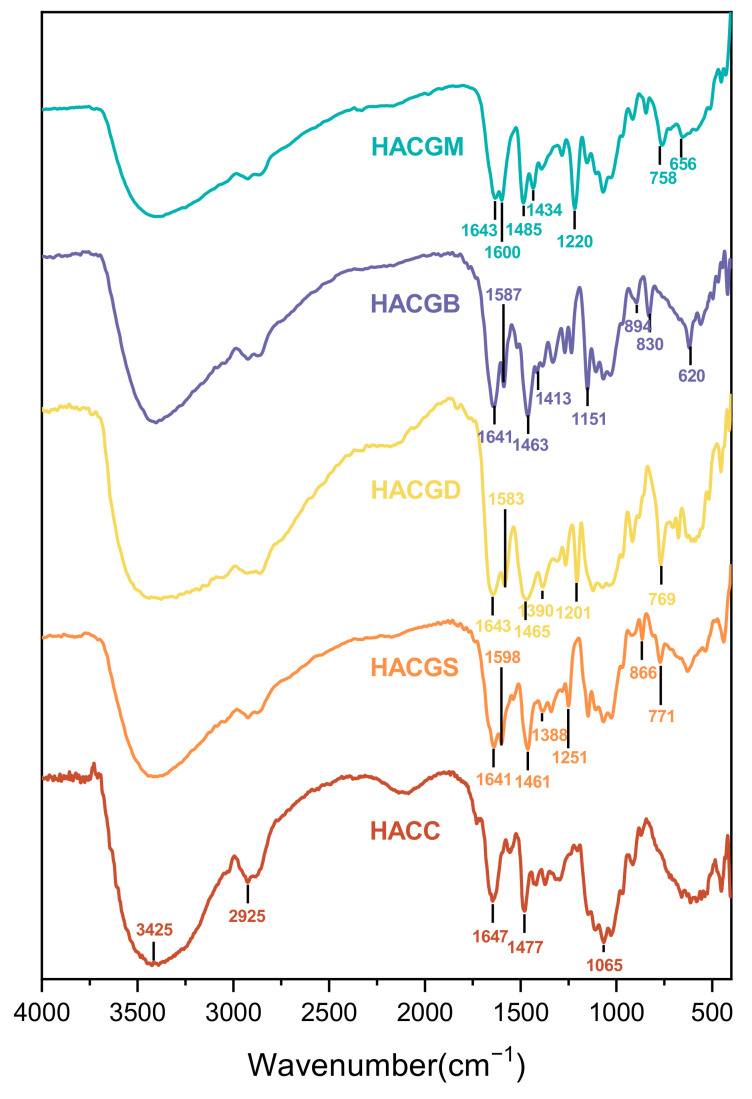
Fourier Transform Infrared (FT-IR) spectra of HACC and HACC derivatives.

**Figure 2:**
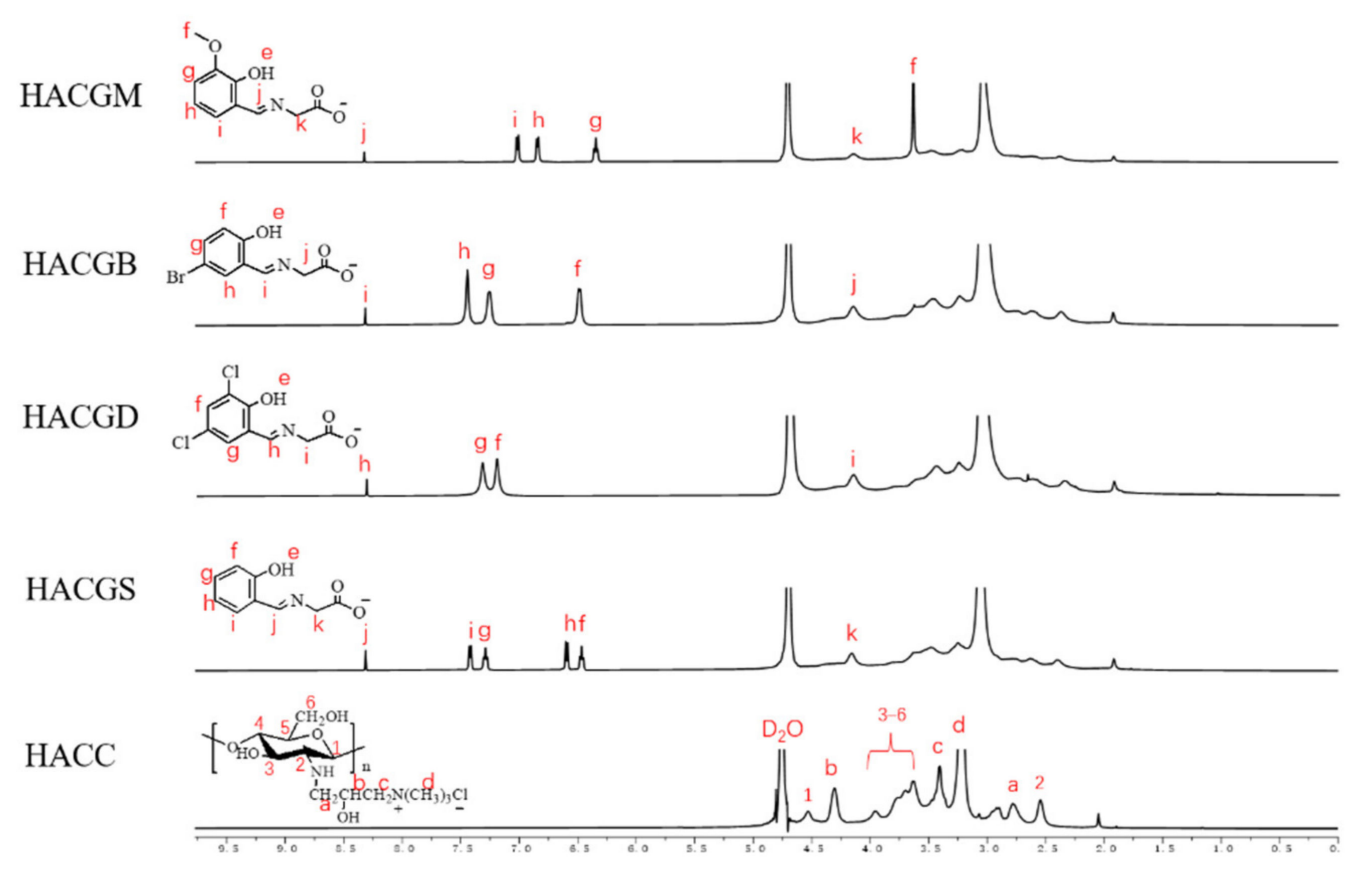
^1^H NMR spectra of HACC and HACC derivatives.

The composition strategy of HACC derivatives is shown in Scheme 1. Firstly, glycine reacted with 3-methoxysalicylaldehyde, 5-bromosalicylaldehyde, 3,5-dichlorosalicylaldehyde, and salicylaldehyde to give the corresponding Schiff base potassium salts. Then, the target compounds were obtained by ion exchange with HACC. After dialysis, the products were frozen in the refrigerator and then freeze-dried in low temperature and vacuum. The structures of HACC derivatives were characterized by FT-IR (Figure 1) and ^1^H NMR (Figure 2).

#### 2.1.1. FT-IR Analysis

The FT-IR spectra of HACC, HACGM, HACGB, HACGD, and HACGS are shown in Figure 1. The absorption peaks of HACC appeared in 3425 cm^−1^ (O-H and N-H stretching vibration), 2925 cm^−1^ (C-H of sugar rings), and 1647 cm^−1^ (C=O of amide bond), and the absorption peak of the trimethylammonium group appeared at 1477 cm^−1^ after quaternization of chitosan [37]. For HACGM, HACGB, HACGD, and HACGS, a new absorption peak, different from HACC, appeared near 1640 cm^−1^ with moderate intensity and was attributed to the C=N stretching vibration peak of the Schiff bases. Additionally, there a new peak appeared at about 1460 cm^−1^, which was assigned to the absorption peak of the trimethylammonium group. The asymmetric stretching vibration and symmetric stretching vibration peaks of carboxyl group appeared approximately at 1600 cm^−1^ and 1400 cm^−1^, respectively. In addition, For HACGM, the stretching vibration of Ar-O-C appeared at 1220 cm^−1^. For HACGB, the C-Br stretching vibration strong absorption peak showed at 620 cm^−1^. For HACGD, the C-Cl stretching vibration strong absorption peak showed at 769 cm^−1^. The further structural data of those compounds were interpreted by ^1^H NMR [38,39,40].

#### 2.1.2. H NMR Analysis

As shown in Figure 2, in the spectrum of hydrogen atoms located in the HACC frame, there was an obvious characteristic absorption of hydrogen (3.10 ppm) of trimethylammonium groups [41]. The absorption of hydrogen atoms in the benzene ring of HACC derivatives was labeled in graphs. Those hydrogen atoms located in the compound imine showed absorptions at around 8.30 ppm; hydrogen atoms connected with carboxyl-bonded carbon atoms showed absorptions around 4.15 ppm [42]. Information about the other hydrogens is as follows:

HACGM: ^1^H NMR (500 MHz, D_2_O): δ 8.31 ppm (s, 1H, Ar-CH=N); δ 7.01 ppm (s, 1H, Ar-H_i_); δ 6.84 ppm (s, 1H, Ar-H_h_); δ 6.35 ppm (s, 1H, Ar-H_g_); δ 4.14 ppm (s, 2H, -CH_2_-); δ 3.63 ppm (s, 3H, Ar-O-CH_3_).

HACGB: ^1^H NMR (500 MHz, D_2_O): δ 8.32 ppm (s, 1H, Ar-CH=N); δ 7.45 ppm (s, 1H, Ar-H_h_); δ 7.27 ppm (s, 1H, Ar-H_g_); δ 6.50 ppm (s, 1H, Ar-H_f_); δ 4.15 ppm (s, 2H, -CH_2_-).

HACGD: ^1^H NMR (500 MHz, D_2_O): δ 8.32 ppm (s, 1H, Ar-CH=N), δ 7.83 ppm (s, 1H, Ar-H_g_), δ 7.21 ppm (s, 1H, Ar-H_f_), δ 4.16 ppm (s, 2H, -CH_2_-).

HACGS: ^1^H NMR (500 MHz, D_2_O): δ 8.32 ppm (s, 1H, Ar-CH=N); δ 7.43 ppm (s, 1H, Ar-H_i_); δ 7.29 ppm (s, 1H, Ar-H_g_); δ 6.61 ppm (s, 1H, Ar-H_h_); δ 6.47 ppm (s, 1H, Ar-H_f_); δ 4.19 ppm (s, 2H, -CH_2_-).

HACC: ^1^H NMR (500 MHz, D_2_O): δ 4.53 ppm (s, 1H, pyranose rings-H_1_); δ 4.31 ppm (s, 1H, H_b_); δ 4.0–3.5 ppm (5H, pyranose rings-H_3-6_); δ 3.41 ppm (s, 2H, H_c_); δ 3.22 ppm (s, 9H, H_d_); δ 2.77 ppm (s, 2H, Ha); δ 2.54 ppm (s, 1H, pyranose rings-H_2_).

By integrating and calculating, the DS of the four compounds was HACGM (54%), HACGB (54%), HACGD (57%), and HACGS (43%).

### 2.2. Antioxidant Capacity

**Figure 3:**
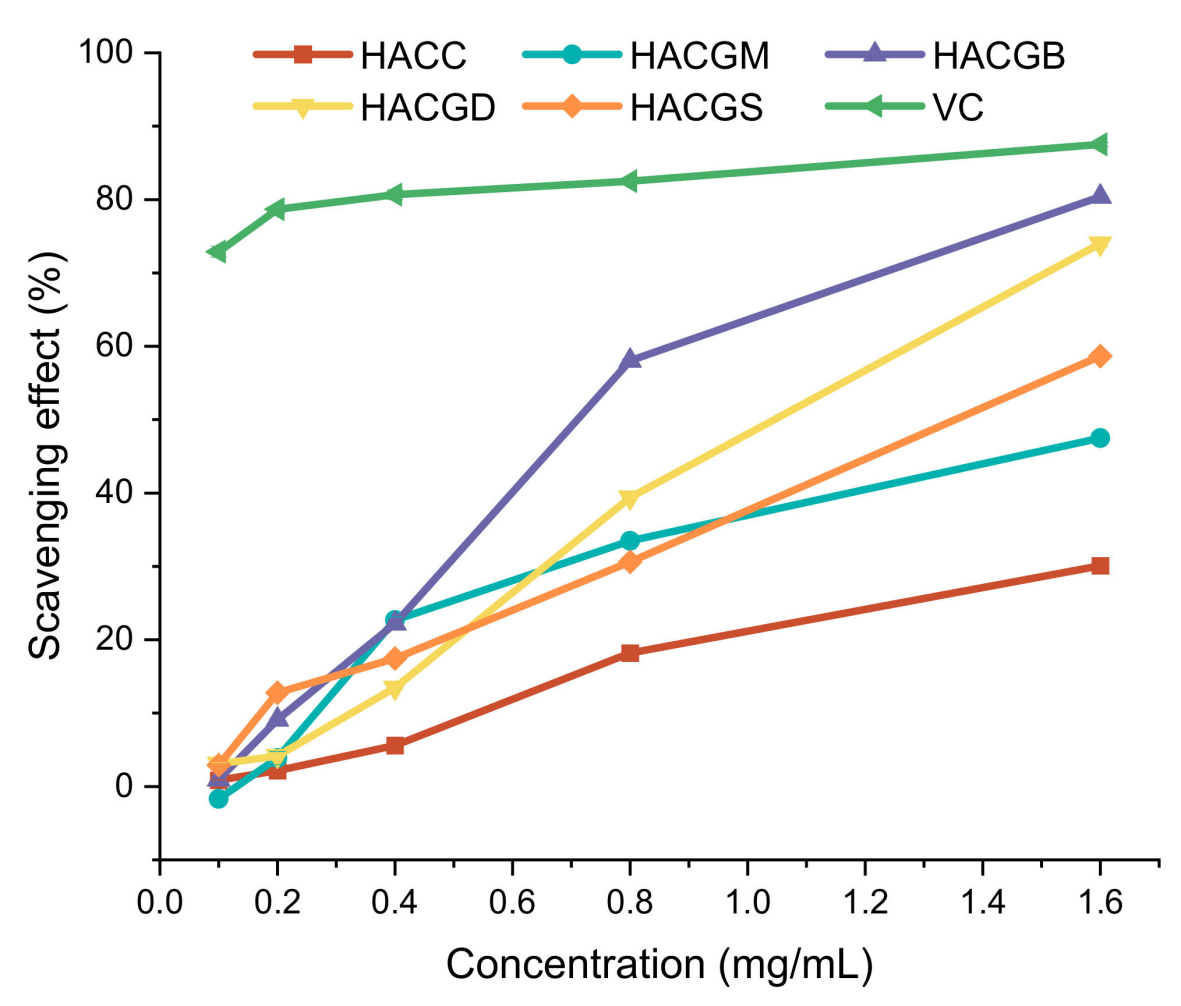
The superoxide radical-scavenging capacity of HACC and HACC derivatives.

In the metabolism of living organisms, oxygen in cells can produce reactive oxygen species (ROS) in mitochondria, including superoxide anion (O_2_^−^), hydrogen peroxide (H_2_O_2_), hydroxyl radical (OH−), ozone (O_3_), and singlet oxygen (^1^O_2_) [43]. They have high chemical reaction activity because of unpaired electrons. One of the electron products of oxygen, in particular, superoxide anion (O_2_^−^), has strong oxidation and reduction ability. When large quantities of O_2_^−^ accumulate in the human body, they can destroy cell membranes and harm tissue metabolism. As can be seen from Figure 3, after modification, the superoxide radical-scavenging capacity of the compounds was improved, especially for HACGB, whose scavenging ability reached 80.32% at the concentration of 1.6 mg/mL, followed by HACGD, which reached 74.01%. With the increase of concentration, the scavenging ability gradually showed a certain trend: HACGB > HACGD > HACGS > HACGM > HACC.

**Figure 4:**
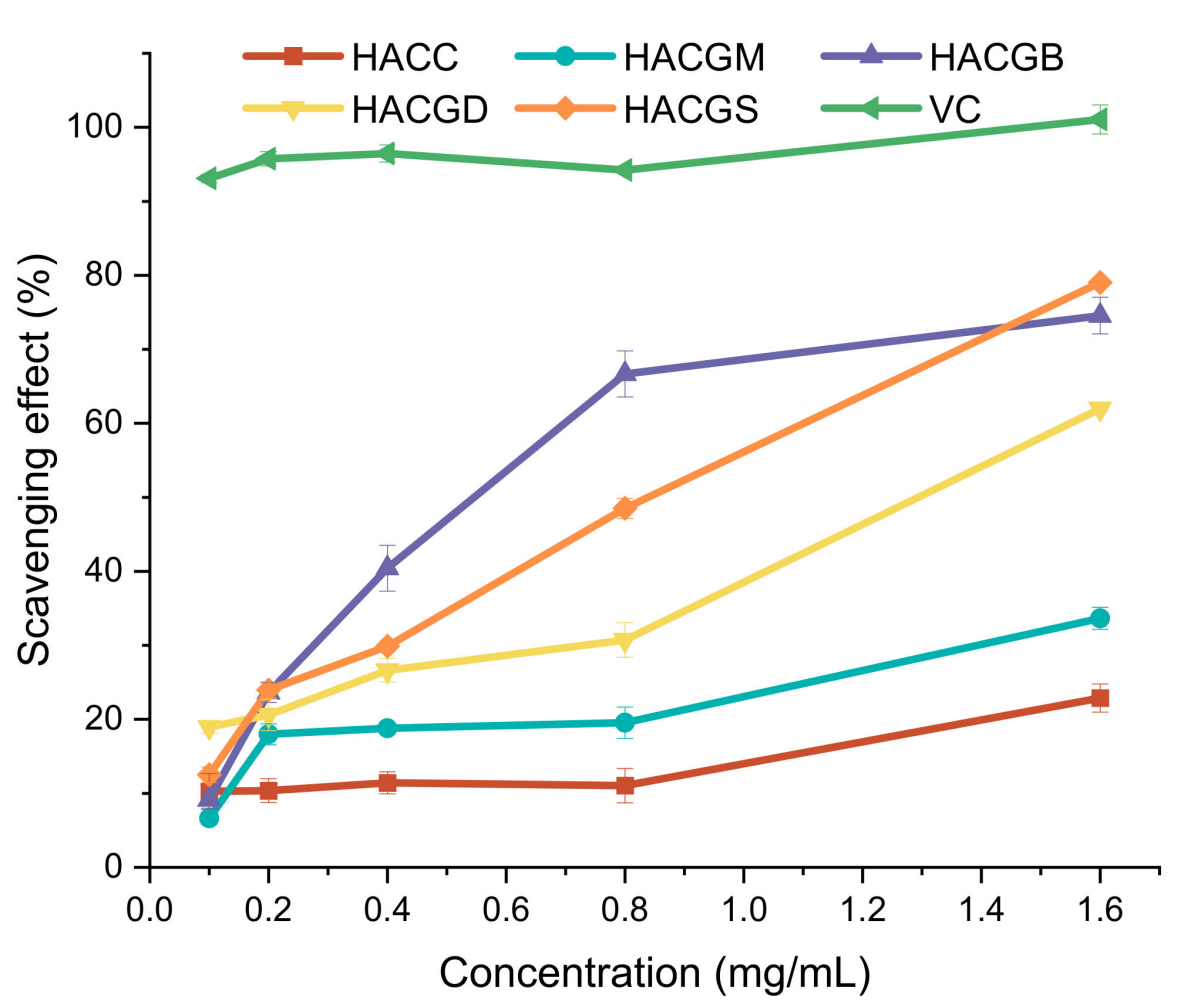
The DPPH-radical scavenging capacity of HACC and HACC derivatives.

DPPH (1,1-diphenyl-2-trinitrophenylhydrazine) is a very stable nitrogen center free radical. Under the action of resonance stabilization, steric hindrance generated by three benzene rings prevents the two lone pair electrons on nitrogen atoms from being uncoordinated [44]. When the solution with free radical scavenging ability was added, lone pair electrons were paired. The dark purple DPPH free radical was reduced to the yellow DPPH-H non-free radical form. Because of its maximum absorbance at 517 nm, the following results were obtained by measuring the change of absorbance. As shown in Figure 4, When the concentration was less than 0.8 mg/mL, the scavenging ability of HACC and HACGM did not change anymore. However, the scavenging ability of HACGS changed obviously with the increase of concentration. On the whole, the DPPH radical scavenging activity of the five compounds was as follows: HACGB > HACGS > HACGD > HACGM > HACC at the concentration of 0.2 mg/mL to 1.4 mg/mL.

### 2.3. Antibacterial Activity

**Figure 5:**
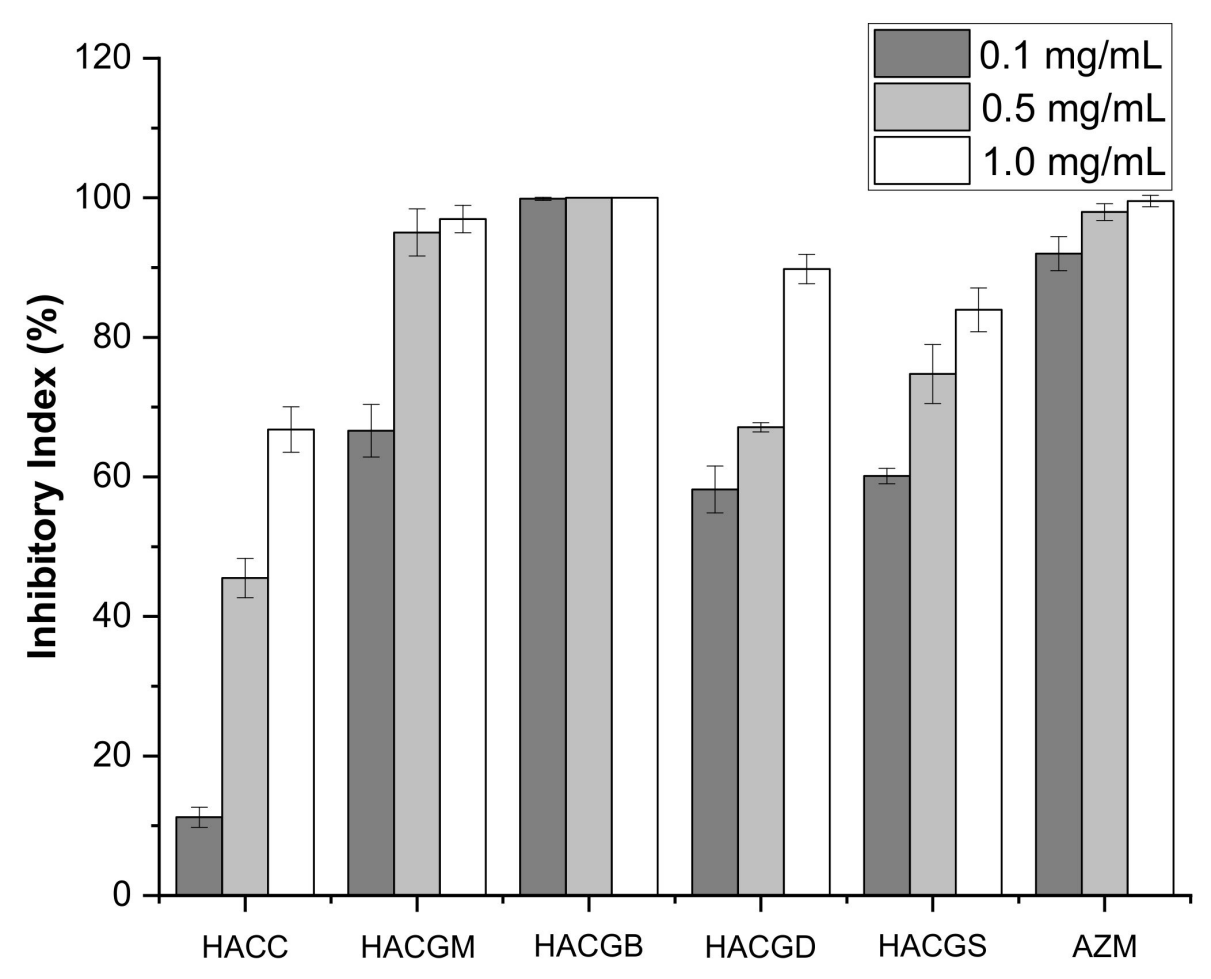
The antibacterial activity of HACC and HACC derivatives against *S. aureus*.

**Figure 6:**
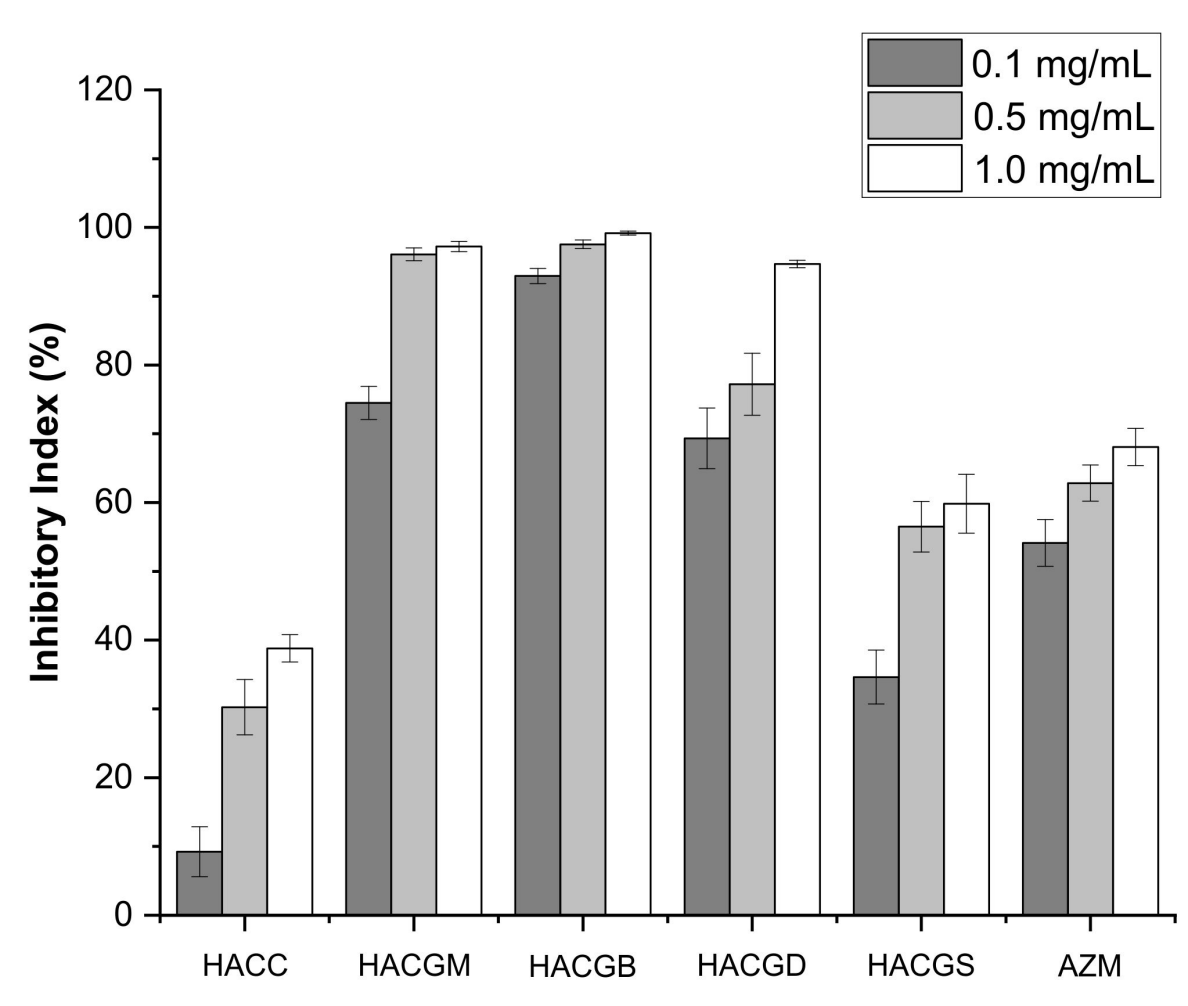
The antibacterial activity of HACC and HACC derivatives against *E. coli*.

The antibacterial activity of four compounds against *S. aureus* and *E. coli* was determined by the plate counting method, and it showed better antimicrobial efficacy compared with HACC. As shown in Figure 5, at concentrations of 0.1 mg/mL, 0.5 mg/mL, and 1.0 mg/mL, the inhibition rates of HACC against *S. aureus* were 11.21%, 45.50%, and 66.80%, respectively. As shown in Figure 6, at concentrations of 0.1 mg/mL, 0.5 mg/mL, and 1.0 mg/mL, the inhibition rates of HACC against *E. coli* were 9.22%, 30.25%, and 38.79%, respectively. After modification, the antibacterial activity of the compounds was greatly improved. HACGB possessed the strongest inhibitory ability on the two bacteria, followed by HACGM. HACGB showed 99.87% and 92.95% rates of inhibition against *S. aureus* and *E. coli* at the concentration of 0.1 mg/mL, respectively, HACGM possessed the antibacterial activity of 74.48% and 66.61% of *S. aureus* and *E. coli* at the concentration of 0.1 mg/mL, respectively. Consistent with their previous studies, the inhibitory ability of these compounds on *S. aureus* was better than on *E. coli*. Based on the structure–activity relationship, they proposed hypotheses on the antibacterial mechanism of chitosan quaternary ammonium salt. The positive charge of chitosan quaternary ammonium salt can produce electrostatic interaction with the negative charge on the surface of microorganisms. Then, cell permeability changes, osmotic balance is destroyed, and the growth of microorganisms is limited [45]. However, chitosan quaternary ammonium salt with low molecular weight could cross the cell membrane, enter the nucleus, and combine with DNA, affecting transcription and translation, thus leading to the death of microorganisms.

### 2.4. Antifungal Activity

**Figure 7:**
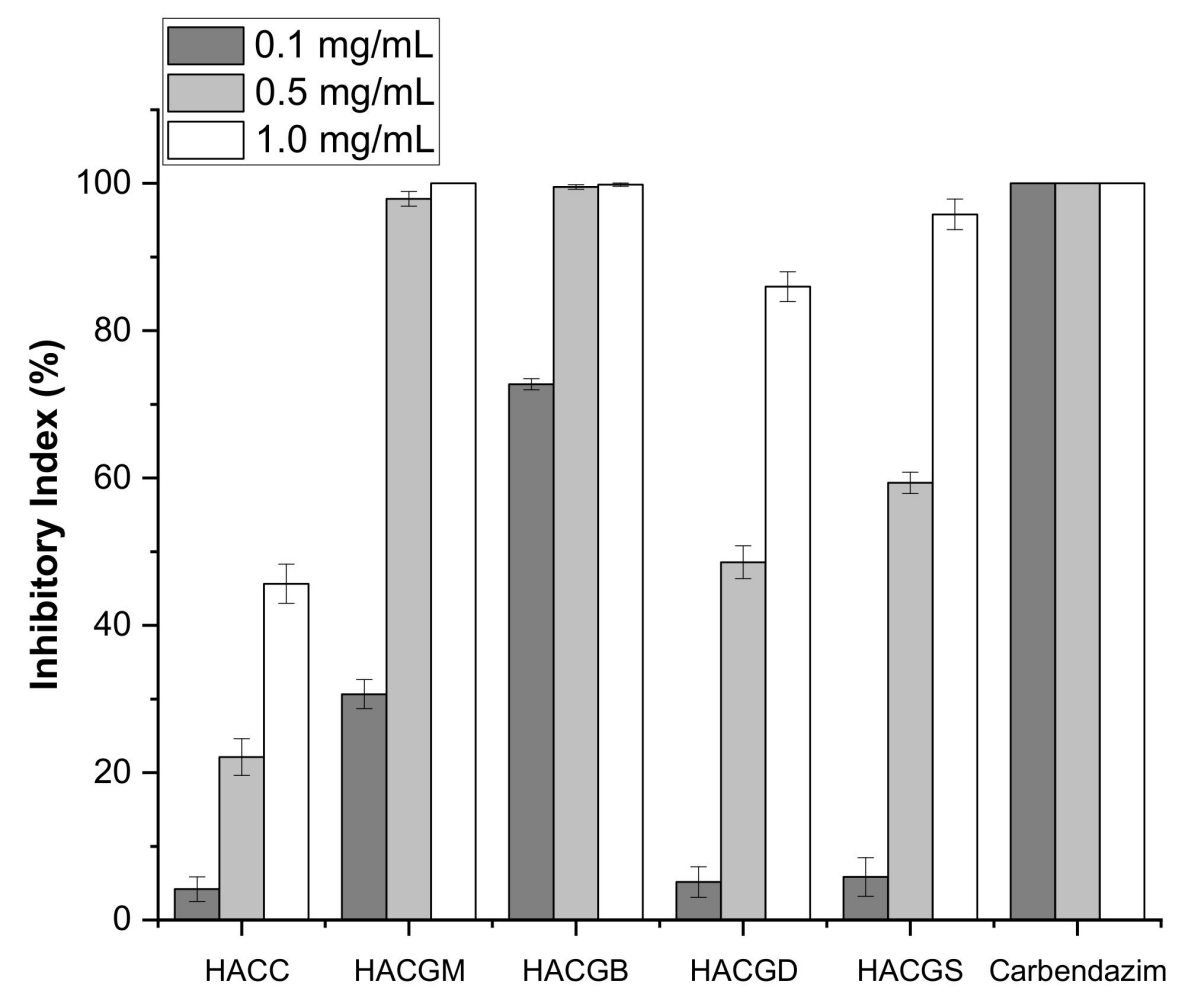
The antifungal activity of HACC and HACC derivatives against *B. cinerea*.

Infecting through multiple pathways, *B. cinerea*, the fungi requiring loose condition to grow, could easily cause plant decay and large-scale crop loss [46]. Therefore, it is of great significance to prepare good bacteriostatic agents. The inhibitory effects of four newly synthesized compounds on *B. cinerea* were determined by mycelium growth rate method. The results showed that all the four compounds had the ability to inhibit *B. cinerea* growth in a concentration-dependent manner. In particular, the antifungal ability of HACGM and HACGB achieved 100%, which was far more than HACC at the concentrations of 0.5 mg/mL and 1.0 mg/mL. Through Figure 7, it was clear that the sequencing of antifungal ability was HACGB > HACGM > HACGS > HACGD > HACC.

**Figure 8:**
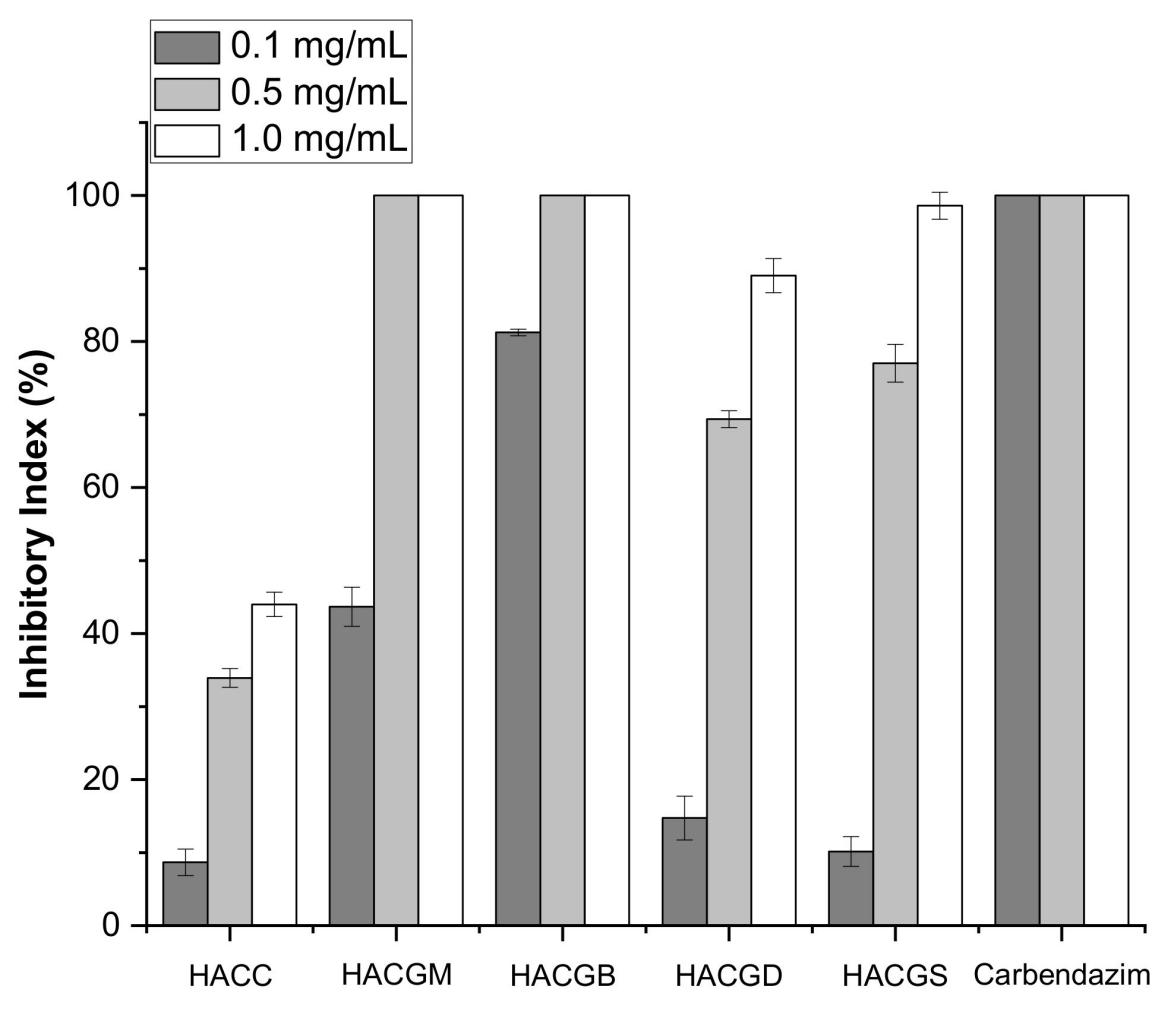
The antifungal activity of HACC and HACC derivatives against *Foc*.

The spores of *Foc* can survive in soil for decades, which brings great danger to the development of the banana industry. The antifungal activity of HACC and HACC derivatives against *Foc* was tested and the results are shown in Figure 8. In particular, HACGM and HACGB can completely inhibit the growth of *Foc* at the concentration of 0.5 mg/mL. HACGD and HACGS also showed good inhibition, with the inhibition rates of 89.04% and 98.60% at the concentration of 0.5mg/mL, respectively. The results above demonstrate that the inhibitory activity of HACC against fungi is not very good. At the concentration of 1.0 mg/mL, the inhibitory rates of HACC on *B. cinerea* and *Foc* were 45.64% and 44.00%, respectively. After modification, the inhibitory effect of the four compounds on fungi was greatly improved. In addition, the four modified compounds showed better inhibition of *Foc* than of *B. cinerea*, which seemed to be related to the structures of fungi.

## 3. Materials and Methods

### 3.1. Materials

Glycine and potassium hydroxide were purchased from Sinopharm Chemical Reagent Co., Ltd., Shanghai, China. Ethanol absolute was obtained from Fuyu Fine Chemicals Co., Ltd., Tianjin, China. Chitosan with a molecular weight of 2.0 × 10^4^ and 94% *N*-deacetylation were purchased from Golden-Shell Pharmaceutical Co., Ltd. (Zhejiang, China). The 3-Methoxysalicylaldehyde, 5-Bromosalicylaldehyde, 3,5-Dichlorosalicylaldehyde, and Salicylaldehyde were purchased from Merck Life Science (Shanghai) Co., Ltd., Shanghai, China. Chloramphenicol and azithromycin were purchased from Aladdin Biochemical Technology Co., Ltd., Shanghai, China.

### 3.2. Synthesis of HACC Derivatives

#### 3.2.1. Synthesis of HACC

The chitosan (3.22 g, 20 mmol) was uniformly dispersed in isopropanol (160 mL) by mechanical agitation. Then, 8 mL of sodium hydroxide solution (40%, *w/v*) were added to the flask and stirred at 60 °C for 4 h. The 3-chloro-2-hydroxypropyl trimethyl ammonium chloride solution (25 mL, 60%) was added to the system drop by drop after stirring and the flask was kept at 80 °C for 10 h. Diluted hydrochloric acid was used to balance the pH to neutral, which was described by a previous study [37]. Finally, HACC was obtained by precipitation, filtration, ethanol washing, and vacuum drying.

#### 3.2.2. Synthesis of Glycine Schiff Base Potassium Salt

Ten mmol of potassium hydroxide and 10 mmol of glycine were dissolved in 50 mL of anhydrous ethanol with continuous stirring at 50 °C. After potassium hydroxide and glycine were completely dissolved, the solution was dropped in anhydrous ethanol solution containing 10 mmol of aldehyde. The types of aldehydes were 3-methoxysalicylaldehyde, 5-bromosalicylaldehyde, 3,5-dichlorosalicylaldehyde, and salicylaldehyde.

#### 3.2.3. Synthesis of HACC Derivatives

One portion of aqueous solution containing HACC (0.31 g, 0.001 mol) was mixed with another portion of an aqueous solution containing amino acid Schiff base potassium salt (0.002 mol). The mixture was stirred at room temperature for 6 h and then placed in a dialysis bag with an interception molecular weight of 500 kDa for 72 h. During this time, small molecules were removed by constantly changing distilled water.

### 3.3. Analytical Methods

#### 3.3.1. Fourier Transform Infrared (FT-IR) Spectroscopy

Fourier transform infrared (FT-IR) spectroscopy, named Nicolet iS50 Fourier Transform Infrared Spectrometer (Thermo, USA, provided by Thermo Fisher Scientific, Shanghai, China) was used to analyze these compounds’ structures at a 4000–400 cm^−1^ spectral range. Samples were mixed with potassium bromide and ground into thin slices.

#### 3.3.2. H Nuclear Magnetic Resonance (NMR) Spectroscopy

The ^1^H NMR spectra were collected on a Bruker AVIII-500 Spectrometer (Switzerland, provided by Bruker Tech. and Serv. Co., Ltd. Beijing, China) operated at a resonance frequency of 500 MHz. The degrees of substitution (DS) of the derivatives were calculated according to ^1^H NMR spectra. For example, the DS of HACGM were measured by the integral ratio of the *H_d_* of HACC and *H_g_* of HACGM. The formula is as follows:

(1) $$\mathrm{DS}\;\left(\%\right)=\frac{{9\;\times \;I}_{H_{g}}}{I_{H_{d}}}$$

where $I_{H_{g}}$ is the integral values of the *H_g_* of HACGM, $I_{H_{d}}$ is the integral values of the *H_d_* of HACC, and nine is the number of protons in *H_d_* of HACC.

### 3.4. Antioxidant Assay

#### 3.4.1. Superoxide Radical Scavenging Ability Assay

Phenazine methyl sulfate (PMS) underwent redox reaction with reduced coenzyme I (NADH) in a slightly alkaline solution to produce a superoxide anion radical (O_2_^−^). The superoxide anion radical (O_2_^−^) was combined with Nitroblue Tetrazolium Chloride (NBT), and the solution was blue. If the compound under testing had antioxidant activity, the superoxide anion radical (O_2_^−^) was first bound to the antioxidant and the solution faded. The superoxide radical scavenging ability was determined according to Nishikimi et al. [47]. The reaction mixture, containing HACC derivatives, PMS (30 μM), NADH (338 μM), and NBT (72 μM) in Tris-HCl buffer (16 mM, pH 8.0), was reacted at room temperature for 5 min and the absorbance was measured at 560 nm against a blank. The capability of scavenging superoxide radical was calculated using the following equation:

(2) $$\mathrm{Scavenging}\;\mathrm{effect}\;\left(\%\right)=\left(1\;-\;\frac{A_{experimental}{\;-\;A}_{\mathrm{control}}}{A_{\mathrm{blank}}}\right)\times 100$$

where *A_experimental_* is the absorbance of the experimental group at 560 nm, A_control_ is the absorbance of the control group at 560 nm, and A_blank_ is the absorbance of the control group at 560 nm.

#### 3.4.2. DPPH Radical Scavenging Ability Assay

This test followed the previous method [48]. The samples to be tested were added to deionized water in different containers and the concentrations of solutions were 0.3 mg/mL, 0.6 mg/mL, 1.2 mg/mL, 2.4 mg/mL, and 4.8 mg/mL. At each concentration level, solutions were divided into three groups, serving as the blank group, experimental group, and the control group. Then, 2.0 mL of deionized water were added to each sample of the blank group, 2.0 mL of DPPH ethanol solution was added to each sample of the experimental group, and 2.0 mL of anhydrous ethanol solution was added to the control group. The absorbance of the experimental group, control group, and blank group was measured at 517 nm. Finally, the above experimental steps were repeated three times and the DPPH-radical scavenging ability assay of the sample was as follows:

(3) $$\mathrm{Scavenging}\;\mathrm{effect}\;\left(\%\right)=\left(1\;-\;\frac{A_{experimental}{\;-\;A}_{\mathrm{control}}}{A_{\mathrm{blank}}}\right)\times 100$$

where *A_experimental_* is the absorbance of the experimental group at 517 nm, A_control_ is the absorbance of the control group at 517 nm, and A_blank_ is the absorbance of the blank group at 517 nm.

### 3.5. Antibacterial Assay

The bacteriostatic activity against *E. coli* and *S. aureus* (10^5^ CFU/mL) of HACC derivatives was determined by the flat colony counting method [49]. Firstly, a solid medium was prepared with a formula ratio of tryptone: yeast extract powder:agar powder:sodium chloride:sterile water = 10:5:18:10:1000. After high-pressure steam sterilization, the solid medium was mixed with the sample in different proportions and finally prepared into 0.1 mg/mL, 0.5 mg/mL, and 1.0 mg/mL medium solutions, which were poured into the mixture in sterile petri dishes and cooled until solidification. Then, 0.1 mL of the revitalized and diluted bacterial solution was absorbed and evenly spread on the surface of solid medium with a coating stick. The bacterial plates grew at 37 °C for 24 h and the colony number was measured. The bacteriostatic rates were calculated as follows:

(4) $$\mathrm{Bacteriostasis}\;\mathrm{Rate}\;\left(\%\right)=\left(1\;-\;\frac{A_{sample}}{A_{\mathrm{blank}}}\right)\times 100$$

where *A_sample_* is the colony number of the sample to be tested and A_blank_ is the colony number of the blank sample. All experiments were repeated three times.

### 3.6. Antifungal Assay

The inhibitions of HACC derivatives on plant fungi (*B. cinerea* and *Foc*) were measured by the mycelium growth rate method [50]. All samples were dried to constant weight and sterile water was added to prepare sample solutions of 5 mg/mL. Prepared sample solutions of 0.3 mL, 1.5 mL, and 3.0 mL were added to the medium of 14.7 mL, 13.5 mL, and 12.0 mL, respectively. The final concentrations of sample media were 0.1 mg/mL, 0.5 mg/mL, and 1.0 mg/mL. After that, the media with the above concentrations were poured into sterile petri dishes, and fungi mycelium with a diameter of 5 mm was inoculated into each petri dish. The colony diameters were measured by the criss-crossing method after 3 days of culture at 27 °C. In addition, the positive control was carbendazim at equal concentration and the blank control was sterile water at equal volume. Fungal inhibition rates were calculated as follows:

(5) $$\mathrm{Fungistatic}\;\mathrm{Rate}\;\left(\%\right)=\left(1\;-\;\frac{D_{sample}\;-\;5}{D_{\mathrm{blank}}\;-\;5}\right)\times 100$$

where *D_sample_* is the diameter of the sample medium and D_blank_ is the diameter of the blank medium. All experiments were repeated three times.

### 3.7. Statistical Analysis

All the data in this article were represented as mean ± the standard deviation (SD), *n* = 3. One-way analysis of variance (ANOVA) and multiple comparison test (TUKEY) were used to analyze and compare the data. A significant difference of *p* < 0.05 was considered statistically significant.

## 4. Conclusions

In conclusion, amino acid Schiff bases were inserted into the framework of HACC to prepare new compounds, which improved the biological activity of chitosan quaternary ammonium salt. The accuracy of the structures was verified by FT-IR and ^1^H NMR. Compared with HACC, the scavenging abilities of the DPPH radical and the superoxide radical of the modified compounds were greatly improved. In addition, the inhibition capacity of the four compounds against bacteria and fungi was greater than that of HACC at different concentrations, especially HACGM and HACGB. It is a remarkable fact that the inhibition rate of HACGB to *S. aureus* and *E. coli* could reach 100% at the concentration of 0.1 mg/mL. Additionally, it also showed a good inhibition effect on two kinds of agricultural pathogens, *B. cinerea* and *Foc* at low concentration. In general, HACC derivatives had good antioxidant capacity and also had a good ability to inhibit fungi and bacteria. This discovery provides reference for the further developments and applications of chitosan and its derivatives in agriculture.

## Data Availability

All data contained in the manuscript are available from the authors.
